# Postdelivery Intervention to Prevent Type 2 Diabetes and the Cost-Effectiveness of Screening Criteria for Gestational Diabetes

**DOI:** 10.5888/pcd19.220055

**Published:** 2022-12-29

**Authors:** Simon J. Neuwahl, Andrea J. Sharma, Ping Zhang, Thomas J. Hoerger

**Affiliations:** 1 RTI International, Research Triangle Park, North Carolina; 2 Centers for Disease Control and Prevention, Atlanta, Georgia; 3 US Public Health Service Commissioned Corps, Atlanta, Georgia

## Abstract

**Purpose and Objectives:**

The objective of our study was to model the costs and benefits of 2 screening criteria for people with gestational diabetes. Because people with a history of gestational diabetes are at increased risk for type 2 diabetes, we modeled the effects of a postdelivery intervention based on the Diabetes Prevention Program, which is offered to all people with a history of gestational diabetes defined by either set of criteria.

**Intervention Approach:**

We used a probabilistic decision tree model to compare the cost-effectiveness of the International Association of Diabetes in Pregnancy Study Group’s (IADPSG’s) screening criteria and the Carpenter–Coustan screening criteria for gestational diabetes through delivery and a follow-up period during which people might develop type 2 diabetes after pregnancy.

**Evaluation Methods:**

The model included perinatal outcomes for the infant and mother and a 10-year postdelivery period to model maternal progression to type 2 diabetes. The model assumed the health care system perspective. People with gestational diabetes received treatment for gestational diabetes during pregnancy, and we assumed that 10% would participate in a Diabetes Prevention Program–based postdelivery intervention to reduce the risk of type 2 diabetes. We estimated the cost-effectiveness of each screening strategy in quality-adjusted life-years (QALYs) in 2022 dollars.

**Results:**

At 10% participation in a Diabetes Prevention Program–based postdelivery intervention, the Carpenter–Coustan criteria were cost-effective, compared with no screening ($66,085 per QALY). The IADPSG screening criteria were slightly less cost-effective, compared with no screening ($97,878 per QALY) or Carpenter–Coustan screening criteria ($122,279 per QALY). With participation rates of 23% or higher, the IADPSG screening criteria were highly cost-effective ($48,588 per QALY), compared with Carpenter–Coustan screening criteria.

**Implications for Public Health:**

Diagnosing a larger proportion of pregnant people using the IADPSG screening criteria, compared with using Carpenter–Coustan screening criteria, is not cost-effective at low levels of participation. However, with moderate levels of participation (23%) in a Diabetes Prevention Program–based postdelivery intervention, the expanded IADPSG screening criteria are cost-effective and reach up to 4 times as many people as Carpenter–Coustan screening.

**Summary**

**What is already known on this topic?**

Lifestyle interventions such as the Diabetes Prevention Program can prevent or delay the progression to type 2 diabetes for people with a history of gestational diabetes. However, participation rates in these interventions are typically low.

**What is added by this report?**

We evaluated the cost-effectiveness of screening and treatment of gestational diabetes assuming a modest level of participation in a Diabetes Prevention Program–based postdelivery intervention.

**What are the implications for public health practice?**

All screening criteria for gestational diabetes were cost-effective at low levels of participation in a Diabetes Prevention Program–based postdelivery intervention. The cost-effectiveness of gestational diabetes screening and treatment improves as participation in a Diabetes Prevention Program–based postdelivery intervention increases.

## Purpose and Objectives

During the past 3 decades, definitions of gestational diabetes used in screening tests have evolved. In general, these definitions have become more expansive and have resulted in the diagnosis of at least twice as many pregnant people, predominantly via lower thresholds for blood glucose. Carpenter–Coustan criteria, introduced in 1982, include the administration of a 50-g glucose challenge test to screen out people at low risk of gestational diabetes before the more burdensome screening tests (fasting and 3-hour oral glucose tolerance test) (1). Since the 2010 introduction of the International Association of Diabetes in Pregnancy Study Group’s (IADPSG’s) screening criteria (2), 2 key studies have examined the cost-effectiveness of these screening criteria (3,4). However, the cost-effectiveness of the IADPSG screening criteria is unclear because of the uncertainty about real-world effectiveness and uptake of a postdelivery intervention based on the Diabetes Prevention Program (DPP). Only about 50% of people with a gestational diabetes–affected pregnancy take part in postpartum screening for type 2 diabetes (5), and less than half of people at increased risk for type 2 diabetes participate in recommended prevention programs like a DPP-based postdelivery intervention (6–8). Similarly, real-world lifestyle-change programs like the National DPP (9) report somewhat smaller effects than the highly controlled DPP trial. The National DPP is a Centers for Disease Control and Prevention (CDC)–recognized lifestyle change program based on the DPP trial’s lifestyle intervention.

To better address gestational diabetes screening policy in the context of type 2 diabetes prevention, we incorporated newly available data on real-world lifestyle-change programs similar to a DPP-based postdelivery intervention. Gestational diabetes screening may be cost-effective but only under circumstances that assume follow-up screening for type 2 diabetes risk and a minimum participation level in a DPP-based postdelivery intervention. By modeling real-world scenarios that incorporate these circumstances, we aimed to provide health economic guidance to decision makers still grappling with the question of optimal gestational diabetes screening criteria. Obstetricians, gynecologists, and their health systems can consult our modeling analysis to help determine the likely cost-effectiveness of expanding to IADPSG screening criteria, given their local circumstances.

## Intervention Approach

We constructed a probabilistic decision tree model (the CDC/RTI gestational diabetes policy model) in TreeAge Pro 2019 release 2.1 (TreeAge Software, LLC) to analyze the cost-effectiveness of screening and treatment of gestational diabetes and participation in a DPP-based postdelivery intervention to reduce risk of progression to type 2 diabetes after delivery. The CDC/RTI model compares IADPSG and Carpenter–Coustan screening criteria, which are distinguished by different probabilities for gestational diabetes–associated complications (Table 1). The model includes perinatal outcomes for the infant (admission to neonatal intensive care unit [NICU], macrosomia, shoulder dystocia, brachial plexus injury) and mother (cesarean delivery, preeclampsia/gestational hypertension) and a 10-year postdelivery period to model maternal progression to type 2 diabetes. Evidence was not sufficient to model postdelivery type 2 diabetes outcomes for children. The model assumed the health care system perspective.

**Table 1:** Probabilities of Complications of Gestational Diabetes, by Gestational Diabetes Status, Used in a Study of the Cost-Effectiveness of Screening Criteria for Gestational Diabetes^a^

Complication	Untreated probability, %	Relative risk with treatment	Treated probability, %	Source(s)
**Probability of preeclampsia/gestational hypertension**				
Normal blood glucose level	8.4	—	—	CDC/NCHS 2020 birth data (10)
Meets IADPSG screening criteria for gestational diabetes	10.8	0.63	4.1	CDC/NCHS 2020 birth data; Ethridge et al (11); author assumption^a^
Meets Carpenter–Coustan screening criteria for gestational diabetes	13.6	0.63	8.6	Landon et al (12)
Preexisting type 2 diabetes	20.4	0.63	12.9	CDC/NCHS 2020 birth data (10); Ray et al (13)
**Probability of cesarean delivery^b^**				
Normal blood glucose level	21.9	—	—	CDC/NCHS 2020 birth data (10)
Meets IADPSG screening criteria for gestational diabetes	27.0	0.90	24.3	CDC/NCHS 2020 birth data (10); Ethridge et al (11); author assumption^a^
Meets Carpenter–Coustan screening criteria for gestational diabetes	33.8	0.90	30.4	Landon et al (12)
Preexisting type 2 diabetes	60.2	0.90	54.2	Ray et al (13)
**Probability of admission to neonatal intensive care unit**				
Normal blood glucose level	9.3	—	—	CDC/NCHS 2020 birth data (10); author assumption^a^
Meets IADPSG screening criteria for gestational diabetes	11.8	0.76	7.5^c^	CDC/NCHS 2020 birth data (10); Ethridge et al (11); author assumption^a^
Meets Carpenter–Coustan screening criteria for gestational diabetes	11.7	0.76	8.9	Landon et al (12)
Preexisting type 2 diabetes	35.2	0.76	26.8	Ray et al (13)
**Probability of shoulder dystocia**				
Normal blood glucose level	1.3	—	—	HAPO Study Cooperative Research Group et al (14)
Meets IADPSG screening criteria for gestational diabetes	1.3	0.41	1.3^c^	HAPO Study Cooperative Research Group et al (14); Ethridge et al (11); author assumption^a^
Meets Carpenter–Coustan screening criteria for gestational diabetes	4.0	0.41	1.6	Landon et al (12)
Preexisting type 2 diabetes	5.0	0.41	2.1	Ray et al (13)
**Probability of macrosomia**				
Normal blood glucose level	7.5	—	—	CDC/NCHS 2020 birth data (10)
Meets IADPSG screening criteria for gestational diabetes	10.6	0.42	8.0^c^	CDC/NCHS 2020 birth data (10); O’Sullivan et al (15); author assumption^a^
Meets Carpenter–Coustan screening criteria for gestational diabetes	14.3	0.42	8.0^c^	Landon et al (12)
Preexisting type 2 diabetes	26.5	0.42	11.1	Aljohani et al (16)
**Probability of stillbirth**				
Normal blood glucose level	0.41	—	—	Patel et al (17)
Meets IADPSG screening criteria for gestational diabetes	0.41	1.00	0.41	Patel et al (17)
Meets Carpenter–Coustan screening criteria for gestational diabetes	0.41	1.00	0.41	Patel et al (17)
Preexisting type 2 diabetes	1.59	1.00	1.59	Patel et al (17)

Abbreviations: —, not applicable; CDC, Centers for Disease Control and Prevention; IADPSG, International Association of Diabetes in Pregnancy Study Group; NCHS, National Center for Health Statistics.

a We assumed that 75.5% of people had normal blood glucose levels during pregnancy. This was the residual rate after assuming that 7.8% of pregnant people in the US had Carpenter–Coustan-defined gestational diabetes and that an additional 15.6% had IADPSG-defined gestational diabetes (excluding Carpenter–Coustan–defined gestational diabetes). We also included data for a small group of pregnant people who have preexisting type 2 diabetes during pregnancy (1.1%). IADPSG-defined gestational diabetes refers to gestational diabetes as defined by the expanded screening criteria for gestational diabetes of IADPSG, not Carpenter–Coustan, that is, the incremental group of pregnant people diagnosed by the expanded criteria. Data from Ethridge et al (11) allowed us to define this as an incremental group.

b Assumed primary cesarean delivery rate. The rate would be higher (32.5%) if we assumed the all-cesarean-delivery rate from Landon et al (12) and other sources.

c Instance in which we coded the model to take the maximum of the untreated probabilities for people with normal blood glucose and the treated probability for people with gestational diabetes. Because we found that some treated probabilities of macrosomia and preeclampsia/gestational hypertension dropped below the untreated probabilities for pregnant people with normal blood glucose, on average, we added a “Max()” function to these treated probabilities in the TreeAge model of gestational diabetes. This means that we took the maximum probability, choosing between the normal untreated probability and the gestational diabetes treated probability. For other complications, such as cesarean delivery, it is possible in simulations that a few outlier draws could have resulted in this same problem; however, because we did not observe this problem on average in the internal validation step (as we did for macrosomia and preeclampsia/gestational hypertension), we assumed that this was a realistic representation of gestational diabetes treated/untreated probabilities. In other words, a single woman (represented by 1 simulation) with Carpenter–Coustan-defined gestational diabetes who is treated for gestational diabetes may have a lower probability of cesarean delivery than a woman with normal blood glucose who was untreated, but on average, pregnant people with normal blood glucose have a lower probability of cesarean delivery.

The design for the CDC/RTI model was influenced by previous US models of gestational diabetes (3,4). We expanded on the existing models by incorporating updated data on gestational diabetes complications and new literature on the effectiveness of DPP-based interventions and the willingness of people to participate in these interventions. The DPP-based postdelivery intervention is a real-world type 2 diabetes prevention intervention like the National DPP (9) or the Balance After Baby Intervention (18). Another key addition to the model is the use of a participation rate for the DPP-based postdelivery intervention and a 10-year postdelivery period that captures the costs and outcomes associated with type 2 diabetes that are potentially preventable. The postdelivery period is particularly important because of the substantial cost and quality-of-life effects of type 2 diabetes (19) and the well-documented increases in type 2 diabetes risk associated with a history of gestational diabetes (20,21).

The natural history of gestational diabetes required us to model the ordered, interrelated nature of several gestational diabetes complications. We constructed a probabilistic decision tree model that incorporates the interrelatedness of these complications and reflects the ordering of complications, beginning with preeclampsia (often occurring in the middle to late stages of pregnancy) and continuing through perinatal complications and the onset of postdelivery type 2 diabetes.

### Evaluation methods

We conducted a probabilistic sensitivity analysis by defining all base-case parameters as a distribution and generating 10,000 randomly drawn parameter sets to use in model calculation. Our results represent the average of all 10,000 calculations of the model. All results on costs and quality-adjusted life-years (QALYs) were discounted by 3%. We also included clinical outcomes such as preeclampsia per 100,000 pregnancies for each screening scenario being compared. We calculated an incremental cost-effectiveness ratio (ICER) to compare screening strategies. The ICER is calculated as the difference in costs divided by the difference in QALYs. We considered an intervention cost-effective if the ICER was less than $150,000 per QALY (22,23).

We also conducted sensitivity analyses on 11 key parameters within the reasonable range of values for each parameter. We ranked the results of each sensitivity analysis in a diagram to demonstrate the relative effect of a range of parameter values on the ICER. We also conducted a threshold analysis on the rate of participation in the DPP-based postdelivery intervention. This threshold analysis allowed us to see what rate of participation would be required for a highly cost-effective intervention (an ICER less than $50,000) when comparing IADPSG screening criteria with Carpenter–Coustan screening criteria.

The CDC/RTI model includes only complications that are modifiable by treatment for gestational diabetes or suspected to be modifiable according to the US Preventive Services Task Force report on gestational diabetes treatment outcomes (24). For complications with insufficient evidence or low levels of evidence according to the report, we erred on the side of inclusion if the associated costs (cesarean delivery) or potential quality-of-life effects (NICU admission) were substantial. Some complications with a low level of evidence and a small impact on costs or quality of life were excluded (neonatal hyperbilirubinemia and hypoglycemia).

We assigned a probability to all included perinatal complications on the basis of evidence from the literature. In general, we assumed a monotonic increase in complication probabilities moving from 1) normal blood glucose levels during pregnancy to 2) meeting IADPSG screening criteria to 3) meeting Carpenter–Coustan screening criteria to 4) meeting type 2 diabetes screening criteria (Table 1). This assumption is consistent with evidence from the Hyperglycemia and Adverse Pregnancy Outcome (HAPO) study (14,25). In our model, people with IADPSG-defined or Carpenter and Coustan–defined gestational diabetes were eligible to be assigned gestational diabetes treatment effects. Risk reduction effects (Table 1) were determined by a meta-analysis (26) that relied largely on a trial of treatment for mild gestational diabetes (12) and the Australian Carbohydrate Intolerance Study in Pregnant Women trial (27). Risk reduction effects were assumed to be the same for IADPSG and Carpenter–Coustan screening criteria. When we refer to people with IADPSG-defined gestational diabetes we are referring to people with only IADPSG-defined gestational diabetes and not Carpenter and Coustan–defined gestational diabetes. That is, people with IADPSG-defined gestational diabetes are the incremental group of people diagnosed by the expanded screening criteria of IADPSG.

### Prevalence of gestational diabetes

In our model, people with normal blood glucose levels during pregnancy are screened and are assigned screening costs, but they do not receive any treatment or reduction in complication risks as the other groups do (Table 1). We assumed that 75.5% of people had normal blood glucose levels during pregnancy. This was the residual rate after assuming that 7.8% of pregnant people in the US had Carpenter and Coustan–defined gestational diabetes and that an additional 15.6% had IADPSG–defined gestational diabetes (excluding Carpenter and Coustan–defined gestational diabetes). We also included data for a small group of pregnant people who had preexisting type 2 diabetes during pregnancy (1.1%). We assumed that pregnant people with preexisting type 2 diabetes are diagnosed and treated according to the appropriate standards of care under either screening strategy; thus, they were assumed to have no effect on study results.

We chose prevalence estimates for IADPSG-defined and Carpenter and Coustan–defined gestational diabetes according to a review of US sources. We assumed that the gestational diabetes rate reported in a recent National Vital Statistics Report (28) based on 2020 birth certificate data (7.8%) was representative of Carpenter and Coustan–defined gestational diabetes nationally. According to this census of US births, gestational diabetes increased from 6.0% in 2016 to 7.8% in 2020 (28). We considered using data based on laboratory-confirmed cases of gestational diabetes, but no such data were nationally representative. We assumed that IADPSG-defined gestational diabetes (inclusive of Carpenter and Coustan–defined gestational diabetes) is 3 times more prevalent than Carpenter and Coustan–defined gestational diabetes. We chose a multiplier of 3.0 because it resulted in an overall prevalence of IADPSG-defined gestational diabetes that was slightly higher than the average prevalence of IADPSG-defined gestational diabetes in the US-only HAPO sites reported by Sacks et al (29). The slightly higher rate assumes an increase in gestational diabetes since the HAPO study, but it allows for the possibility that approximately half of the increase was due to some health care providers shifting to IADPSG screening criteria. 

### Screening, treatment, and complication costs

The model included several key gestational diabetes screening, treatment, and complication costs (Table 2). All costs were in 2022 dollars and were inflated by using Personal Consumption Expenditures data for health care services, a price index from the US Bureau of Economic Analysis that measures inflation in the cost of health care services. The cost of normal prenatal care was applied to all pregnant people in the group with normal blood glucose levels during pregnancy and to all pregnant people with type 2 diabetes who were not treated for their condition. Pregnant people treated for gestational diabetes were assigned higher prenatal care costs to reflect the costs of additional office visits, medical nutrition therapy, and in some cases, insulin treatment (30). We assumed that 12% of pregnant people with IADPSG-defined or Carpenter and Coustan–defined gestational diabetes would require insulin treatment (34) and would incur additional prenatal care costs of $591 (30). Extra costs for insulin-treated pregnant people included insulin supplies, additional neonatal heart rate tests, ultrasounds, and some outpatient monitoring (30). Perinatal complication costs were included for NICU admissions and 3-month postdelivery care after vaginal delivery ($7,812) or cesarean delivery ($11,893), preeclampsia or gestational hypertension ($11,052), cesarean delivery ($5,665 incremental cost relative to vaginal delivery), brachial plexus injuries (temporary, $2,774; permanent, $24,786). We also included 10 years of excess medical costs for pregnant people that develop type 2 diabetes during the postdelivery period. Discounted back to the present, these costs totaled $59,300. Shrestha and colleagues estimated the underlying costs in a matched cohort of private health plan patients (19). In year 1 after diagnosis, costs were estimated to be $10,862 per patient. Costs in years 2 through 10 averaged $6,485 per year (Table 2).

**Table 2:** Additional Model Parameters Used in a Study of the Cost-Effectiveness of Screening Criteria for Gestational Diabetes

Model parameter	Value	Source(s)
**Screening costs** a		
IADPSG screening criteria for gestational diabetes (2-h glucose tolerance test)	$32	Medicare Laboratory Fee Schedule 2015
Carpenter–Coustan screening criteria for gestational diabetes		
Glucose challenge test	$12	Medicare Laboratory Fee Schedule 2015, Medicare Physician Fee Schedule 2015
3-Hour glucose tolerance test^b^	$32	
**Prenatal care**		
Normal prenatal care	$653	Moss et al (27)
Prenatal care for gestational diabetes	$1,644	Kitzmiller et al (30)
**Onset of postdelivery type 2 diabetes**		
10-Year risk for onset of type 2 diabetes for people with normal blood glucose level^c^	2.3%	Xiang et al (31); Bellamy et al (21)
10-Year risk for onset of type 2 diabetes for people with gestational diabetes defined by IADPSG screening criteria for gestational diabetes^c^	11.0%	Xiang et al (31); Bellamy et al (21)
10-Year risk of onset for type 2 diabetes for people with gestational diabetes defined by Carpenter–Coustan^c^	17.0%	Xiang et al (31); Bellamy et al (21)
Annual maternal health utility decrement with type 2 diabetes (QALY)	0.11	Alva et al (32)
Annual incremental medical costs for people with type 2 diabetes (first year)^d^	$10,862	Shrestha et al (19)
Annual incremental medical costs for people with type 2 diabetes (years 2–10)^d^	$6,485	Shrestha et al (19)
**DPP-based postdelivery lifestyle intervention**		
Cost of intervention	$632	Li et al (33)
Participation rate^e^	10%	Author assumption
Risk reduction for type 2 diabetes	20.4%	Author assumption

Abbreviations: DPP, Diabetes Prevention Program; IADPSG, International Association of Diabetes in Pregnancy Study Group; NICU, neonatal intensive care unit; OGTT, oral glucose tolerance test, QALY, quality-adjusted life-year.

a All costs shown in 2022 dollars. For all costs, we used payments (ie, not charges) made by a private insurer because Medicaid costs were available for only a few outcomes (cesarean delivery, vaginal delivery, neonatal intensive care). Where Medicaid costs were available, they were about 60% lower than private insurance costs. We assumed that the screening only creates a negligible amount of additional physician time spent with a woman who is already visiting for regular prenatal care.

b Assumes that the OGTT 2-h costs are the same as the OGTT 3-h costs (2-h costs are not published in the Medicare Laboratory Fee Schedule). Physician fees were not included in default model screening costs. We assumed that the screening creates a negligible amount of additional physician time spent with a woman who is already visiting for regular prenatal care.

c Described in detail in Methods section.

d Type 2 diabetes costs for a period of 10 years were applied to all people predicted to develop diabetes during the 10-year postdelivery period. The total present value of these 10-year type 2 diabetes costs was $59,300 in 2022 dollars. Estimates were taken from Shrestha et al (19) and inflated to 2022 dollars.

e The DPP-based postdelivery intervention participation rate was assumed to be 10% in the base case. Participation rates for the real-world version of the DPP have ranged from 43% in the RAPID trial (7) to 5.3% of Medicare Advantage enrollees at risk for type 2 diabetes based on laboratories or claims (6). We conservatively assumed a participation rate of 10% for the DPP-based postdelivery intervention.

Not all complications included in the model were linked directly to medical costs. For example, macrosomia can increase the probability of temporary or permanent brachial plexus injury (Table 2), but it does not have a cost of its own in the model. Because treatment for gestational diabetes reduces the incidence of macrosomia, the risk for brachial plexus injury decreases. Similarly, many perinatal complications do not affect quality of life because they have short-lived effects. Major quality-of-life effects were included for type 2 diabetes, brachial plexus injury, and death. These effects were used to discount a person’s QALY. A QALY of 1.0 is a measure of 1 year of life at full health. QALY values less than 1.0 represent a year of life with less than full health. For example, when someone develops diabetes in the 10-year period after delivery, we reduce their quality of life by 0.11 for all remaining life-years to approximate the health effects of living with type 2 diabetes.

### Postdelivery period

To estimate the onset of type 2 diabetes after delivery, we referred to a retrospective study of pregnant people enrolled in the Kaiser Permanente of Southern California health plan from 1995 through 2009 (31). We also considered using estimates from the National Health Interview Survey (NHIS) (35); however, we found that estimates of type 2 diabetes incidence are typically higher in studies that follow pregnant people after pregnancy (unlike the NHIS) (31), and we wanted our model to reflect this higher risk. The estimated 10-year incidence of type 2 diabetes was 3.0% among pregnant people without a history of gestational diabetes in the Kaiser Permanente study. However, these data were based on a sample of pregnancies from a predominantly Hispanic and Asian population that may not be representative of other US patient populations. Thus, we selected a more conservative 10-year incidence among pregnant people with normal blood glucose levels during pregnancy (2.3%), the rate observed by Xiang et al among non-Hispanic White pregnant people in the Kaiser Permanente study (31).

To estimate the incidence of postdelivery type 2 diabetes for each of the 3 gestational diabetes groups (normal blood glucose, IADPSG-defined gestational diabetes, and Carpenter and Coustan–defined gestational diabetes), we also needed an estimate of the relative risk for type 2 diabetes among people with a history of IADPSG-defined gestational diabetes and Carpenter and Coustan–defined gestational diabetes (relative to people with normal blood glucose levels during pregnancy). We assumed that the relative risk for IADPSG-defined gestational diabetes would be somewhat lower than that for Carpenter and Coustan–defined gestational diabetes. Using data from a 2009 meta-analysis, we assumed a relative risk of type 2 diabetes of 4.8 for IADPSG-defined gestational diabetes and 7.4 for Carpenter and Coustan–defined gestational diabetes (21). Xiang et al showed similar relative risks (31), which were based on the lower bound and base case estimates of Bellamy et al (21). Thus, for our base case, pregnant people with IADPSG-defined gestational diabetes had a risk for type 2 diabetes after delivery of 11.0%, pregnant people with Carpenter and Coustan–defined gestational diabetes had a risk of 17.0%, and pregnant people with normal blood glucose had a risk of 2.3%.

To describe the potential reduction in risk of type 2 diabetes, we included a postdelivery lifestyle intervention in our model. On the basis of 10-year DPP trial outcomes for people with a history of gestational diabetes (36), we calculated an adjusted type 2 diabetes risk reduction of 20.4%. This reflects a proportionally lower risk reduction based on the lower amount of weight loss observed in real-world programs like the National DPP (9) (4.2% of weight lost at last session) compared with programs in a trial setting, such as the DPP (37) (7.2% weight loss at 1 year). We discounted the DPP risk reduction for people with a history of gestational diabetes (35%) by the ratio of National DPP weight loss to DPP trial weight loss (58%) to get an adjusted risk reduction of 20.4%. Not all people receive the benefit of this risk reduction because we assumed that only 10% will participate. Participation rates varied substantially in the literature and ranged from approximately 5% (6) to 41% (7). We set our base case at 10% because of low screening and referral rates observed nationally (8), but we also conducted a sensitivity analysis at higher participation rates. We created a tornado diagram in Excel (Microsoft Corporation) that ranked 11 key parameters from most to least sensitive.

## Results

IADPSG screening criteria were moderately more cost-effective compared with Carpenter and Coustan screening criteria using a willingness-to-pay of $150,000 per QALY (Table 3). At a 10% level of participation in the DPP-based postdelivery intervention, the ICER for IADPSG screening criteria, compared with Carpenter–Coustan screening criteria, was $122,279 per QALY. The IADPSG screening criteria were more cost-effective compared with no screening (ICER, $97,878 per QALY) and the Carpenter–Coustan screening criteria were also more cost-effective compared with no screening (ICER, $66,085 per QALY).

**Table 3:** Incremental Cost-Effectiveness Ratios (ICERs), Cost, and Quality-Adjusted Life-Years (QALYs) for Each Screening Strategy for Gestational Diabetes^a^

Item	Comparison of screening strategies for gestational diabetes			Screening strategy for gestational diabetes		
	Carpenter–Coustan screening criteria vs no screening	IADPSG screening criteria vs no screening	IADPSG screening criteria vs Carpenter–Coustan screening criteria	No screening	Carpenter–Coustan screening criteria	IADPSG screening criteria
Cost per QALY, $	66,085	97,878	122,279	—	—	—
Average cost per pregnancy, $	—			21,255	21,310	21,442
Average no. of QALYs per pregnancy				55.8	55.8	55.8
**Clinical outcomes per 100,000 pregnancies**						
Preeclampsia	—			9,446	8,990	8,375
Macrosomia				8,877	8,185	7,572
Shoulder dystocia				1,228	1,088	1,014
Cesarean delivery				23,821	23,485	23,055
Neonatal intensive care unit				9,972	9,665	9,277
10-Year type 2 diabetes				5,887	5,859	5,822

Abbreviation: —, does not apply; IADPSG; International Association of Diabetes in Pregnancy Study Group.

a Average results for each strategy simulating 10,000 parameter sets to account for parameter uncertainty. Incremental costs and QALYs were calculated relative to the protocol stated second in each comparison. An ICER is the ratio of these incremental costs and QALYs. Lower ICERs are associated with greater cost-effectiveness. ICERs less than $150,000 per QALY are generally considered cost-effective; however, this is just 1 commonly used value for a decision maker’s “willingness-to-pay.”

We found incremental improvements in QALYs gained by implementing IADPSG screening criteria. For each pregnancy, on average, 0.001 QALYs were gained and an additional cost of $132 was incurred by moving from Carpenter–Coustan screening criteria to IADPSG screening criteria (Table 3). Every 100,000 pregnancies resulted in a gain of 100 QALYs and a net screening and treatment cost of $13.2 million, which includes the medical cost savings from preventing or delaying type 2 diabetes.

The modest QALY benefits of IADPSG screening criteria on a per-pregnancy basis (0.001) were largely because approximately 75% of people have normal blood glucose levels during pregnancy and do not receive any benefits from gestational diabetes treatment or the postdelivery intervention.

The sensitivity analysis found that our model was most sensitive to parameters related to type 2 diabetes risk during the postdelivery period: the 10-year risk reduction in type 2 diabetes of the DPP-based postdelivery intervention, followed closely by the participation rate associated with the DPP-based postdelivery intervention, and the 10-year type 2 diabetes risk for people with IADPSG-defined gestational diabetes (Figure). A threshold analysis using the full simulation sample showed that IADPSG screening criteria were more cost-effective than the Carpenter–Coustan screening criteria at postdelivery intervention participation rates of 23% or higher (ICER, $48,588).

**Figure:**
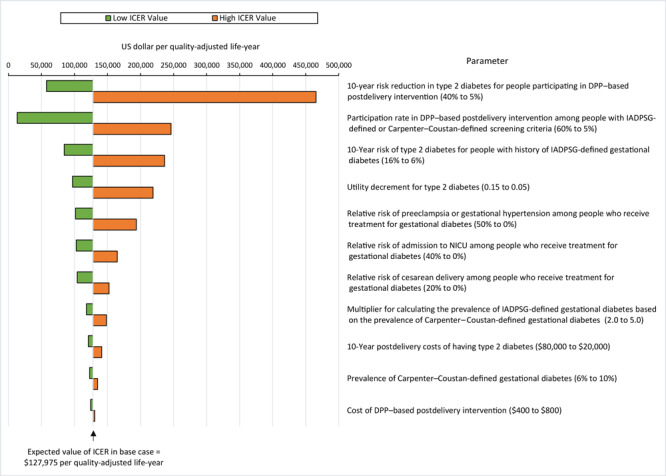
Sensitivity analysis on incremental cost-effectiveness ratios (ICERs) for International Association of Diabetes in Pregnancy Study Group (IADPSG) (2) screening criteria compared with Carpenter–Coustan (1) screening criteria for gestational diabetes. The ICER is calculated as the difference in costs divided by the difference in quality-adjusted life years (QALYs). We considered ICERs cost-effective if they were <$150,000 per QALY. The lower the ICER, the more cost-effective IADPSG criteria compared with Carpenter–Coustan criteria. The figure is centered on the model’s expected ICER value of $127,975, slightly higher than the averaged results across the 10,000 simulations. Values in parentheses are parameter ranges. Abbreviations: DPP, Diabetes Prevention Program; NICU, neonatal intensive care.

**Table:** 

Parameter	Parameter range	Incremental cost-effectiveness ratio, $	
		Low	High
10-year risk reduction in type 2 diabetes for people participating in DPP–based postdelivery intervention	40% to 5%	57,778	465,635
Participation rate in DPP–based postdelivery intervention among people with IADPSG-defined or Carpenter–Coustan-defined screening criteria	60% to 5%	13,442	246,222
10-Year risk of type 2 diabetes for people with history of IADPSG-defined gestational diabetes	16% to 6%	84,384	236,281
Utility decrement for type 2 diabetes	0.15 to 0.05	97,115	218,988
Relative risk of preeclampsia or gestational hypertension among people who receive treatment for gestational diabetes	50% to 0%	101,510	193,614
Relative risk of admission to NICU among people who receive treatment for gestational diabetes	40% to 0%	102,613	164,779
Relative risk of cesarean delivery among people who receive treatment for gestational diabetes	20% to 0%	104,135	152,362
Multiplier for calculating the prevalence of IADPSG-defined gestational diabetes based on the prevalence of Carpenter–Coustan-defined gestational diabetes	2.0 to 5.0	118,030	148,339
10-Year postdelivery costs of having type 2 diabetes	$80,000 to $20,000	120,977	141,262
Prevalence of Carpenter–Coustan-defined gestational diabetes	6% to 10%	122,868	134,940
Cost of DPP-based postdelivery intervention	$400 to $800	124,496	130,492

## Implications for Public Health

In a scenario with low rates of participation in a DPP-based postdelivery intervention (10%), Carpenter–Coustan screening criteria were cost-effective ($66,085 per QALY), compared with no screening, and the expanded IADPSG screening criteria were moderately cost-effective, compared with Carpenter–Coustan screening criteria ($122,279 per QALY). For health care practices that can prompt approximately one-quarter of people with gestational diabetes to participate in the DPP-based postdelivery intervention, the IADPSG screening criteria would be highly cost-effective compared with Carpenter–Coustan screening criteria ($48,588 per QALY). Health care practices and systems can improve participation in a DPP-based postdelivery intervention by ensuring that all patients with a history of gestational diabetes are referred to a DPP-based postdelivery intervention (38).

For many people, gestational diabetes is the beginning of a series of events that eventually lead to type 2 diabetes. By screening for gestational diabetes, we gain the opportunity to identify pregnant people with a higher risk of type 2 diabetes and refer them to a prevention program such as the National DPP or Balance After Baby Intervention. However, this opportunity is often missed as many pregnant people do not even receive postdelivery screening for type 2 diabetes (5).

In health care practices and systems with a high degree of care coordination, such as a patient-centered medical home, the rates of referral to postdelivery screening and patient participation in a DPP-based postdelivery intervention may be higher (38). Our analysis shows that the IADPSG gestational diabetes screening criteria are likely to be cost-effective in these settings. Health care practices and systems with a low degree of care coordination can work to improve patient participation in a DPP-based postdelivery intervention by using tools such as patient reminders for postdelivery screening and formal systems of communication between obstetricians and primary care providers (38,39).

A recent analysis of NHIS data found participation rates of 35% in a weight-loss program and 40% in a DPP-based program when patients diagnosed with prediabetes were referred to such a program by their health care professional (8). The NHIS analysis demonstrated that a participation rate of approximately 40% is achievable for a program similar to a DPP-based postdelivery intervention, and thus the IADPSG screening criteria are likely to be cost-effective incremental to the Carpenter–Coustan screening criteria.

Our model largely validates a previous modeling study by Werner and colleagues (4). However, we diverged in the postdelivery period, where we assumed a lower effectiveness of the DPP-based postdelivery intervention (20% vs 34% reduction in type 2 diabetes risk) and a lower participation rate among people with a history of gestational diabetes (10% vs 100% participation). Research on the effectiveness of a DPP-based postdelivery intervention is still relatively new. Results from the pilot Balance After Baby Intervention showed that it is feasible for new mothers to participate in a DPP-based postdelivery intervention and achieve meaningful weight loss goals (18).

When we compared our findings with those of Mission and colleagues (3), we found a higher ICER for IADPSG screening criteria than for Carpenter–Coustan screening criteria. Even with 10% of people participating in a DPP-based postdelivery intervention, we had an ICER of $122,279 per QALY, whereas Mission and colleagues had an ICER of $61,503 per QALY. The key ways in which the study by Mission and colleagues differed from our study were the lack of a DPP-based postdelivery intervention, a risk reduction effect of approximately 21% (compared with 10% in our study) for cesarean delivery among pregnant people diagnosed according to IADPSG screening criteria (based on the trial of treatment for mild gestational diabetes) (12), and the inclusion of hypoglycemia and hyperbilirubinemia outcomes.

Our model has some limitations. First, recent evidence suggests that gestational diabetes diagnosed according to IADPSG screening criteria may not be applicable to all populations (40). Also, because the IADPSG gestational diabetes screening criteria were developed relatively recently, in 2010, and are not widely used, data are limited on rates of complications attributable to, and risk reductions associated with, treating gestational diabetes diagnosed according to IADPSG screening criteria. However, the parameters used in this study for type 2 diabetes risk (21), cost (19,41), and utility (32) are supported by robust literature and drive much of the results, along with the parameters for DPP-based postdelivery intervention.

Second, we did not include hypoglycemia or hyperbilirubinemia neonatal outcomes. Some evidence suggests that risk of neonatal hypoglycemia increases with macrosomia (42), and we know that gestational diabetes treatment reduces macrosomia; however, the US Preventive Services Task Force did not find any differences in these outcomes with gestational diabetes treatment (24). Third, the model did not include any long-term neonatal outcomes. Recently results from the HAPO Follow-up Study demonstrated a higher risk of childhood impaired glucose tolerance among neonates exposed to maternal IADPSG-defined gestational diabetes than among neonates not exposed (43). However, evidence is still lacking on how neonatal glucose tolerance and other outcomes might be moderated by treatment for people with gestational diabetes.

Referring people with a history of gestational diabetes to a DPP-based postdelivery intervention is recommended by current clinical guidance. The cost-effectiveness of expanded gestational diabetes screening criteria, such as IADPSG screening criteria, improves rapidly with increased participation in a DPP-based postdelivery intervention.
